# Exploiting the behaviour of wild malaria vectors to achieve high infection with entomopathogenic fungus

**DOI:** 10.1186/1475-2875-9-S2-P23

**Published:** 2010-10-20

**Authors:** Ladslaus L Mnyone, Issa N Lyimo, Dickson W Lwetoijera, Monica W Mpingwa, Nuru Nchimbi, Penny Hancock, Tanya L Russell, Matthew J Kirby, Willem Takken, Constantianus JM Koenraadt

**Affiliations:** 1 Biomedical and Environmental Group, Ifakara Health Institute, P.O. Box 53, Off Mlabani Passage, Ifakara, Tanzania; 2 Laboratory of Entomology, Wageningen University and Research Centre, P.O. Box 8031, 6700 EH, Wageningen, The Netherlands; 3 Pest Management Centre, Sokoine University of Agriculture, P.O. Box 3110, Morogoro, Tanzania; 4 Faculty of Biomedical and Life Sciences, University of Glasgow, 120 University Place, G1 2 8TA, Glasgow, UK; 5 Centre for Population Biology, Imperial College London, Silwood Park Campus, Ascot, Berkshire, UK; 6 The University of Queensland, School of Population Health, Australian Centre for Tropical and International Health, Brisbane, 4006, Australia; 7 Vector Group, Liverpool School of Tropical Medicine, Liverpool, L3 5QA, UK

## Background

Control of mosquito vectors has been the mainstay in the fight against malaria, but alternatives are required in view of emerging insecticide resistance. Entomopathogenic fungi have proven to be such an alternative, but to date, very few trials have translated use of these agents to field-based evaluations of their impact on mosquito survival and malaria risk. Delivery techniques that successfully infect mosquitoes need to be developed and tested under field conditions.

## Methods

Mineral oil-formulations of *Metarhizium anisopliae* and *Beauveria bassiana* were applied against wild mosquitoes using five different techniques that each exploited the behaviour of mosquitoes when entering, host-seeking or resting in experimental huts. Techniques employed were eave netting, eave curtains, cloth panels, eave baffles and strips of cotton cloth hung next to bed nets. Three experimental huts were used per trial in which we evaluated one of the five techniques over 9 consecutive nights. Every morning a sub-sample of up to 25 mosquitoes was collected from each hut, placed singly in individual plastic tubes and monitored for survival and fungus infection status. With the obtained data impact of fungal infection on malaria transmission risk was estimated by using a gonotrophic cycle model of mosquito-malaria interactions.

## Results

Few mosquitoes entered huts fitted with eave netting and none of these became infected. Application of fungus on eave curtains and panels did not show any impact on mosquito infection or survival. However, after forcing upward entry of mosquitoes through the eaves (baffle design) or after application of fungus treated surfaces directly on a bed net with a host (strip design), survival of mosquitoes from treatment group was reduced by 6-7 d relative to that of controls. Moreover, 67.9 to 75.5% of treatment mosquitoes showed fungal growth. With effect of fungus on survival alone EIR is estimated to be reduced by 75-80%, whereas EIR is reduced by at least 96% if bed net coverage is ≥0.4 even if mosquitoes are resistant to insecticides and fungus exerts moderate reduction on blood feeding activity.

## Conclusion

The design for delivering fungal conidia to mosquitoes can largely impact on proportion of mosquitoes actually infected and depends on how female mosquitoes behave towards a host. We achieved a fungus infection rate as high as 75% by means of fungus-treated cotton cloth eave baffles/cotton cloth strips hung around bed nets. Our model confirms that such coverage rates are sufficiently large to achieve major reductions in malaria transmission risk.

